# Supplementary material for: Sustained-input switches for transcription factors and microRNAs are central building blocks of eukaryotic gene circuits
Source: Genome Biol. 2013 Aug 23;14(8):R85. doi: 10.1186/gb-2013-14-8-r85 (PMC4054853; doi:10.1186/gb-2013-14-8-r85)
Supplement: Additional file 5 — HTML Browsable Motif Output. Zipped folder containing all WaRSwap and FANMOD motif output, viewable in a web browser. [file gb-2013-14-8-r85-S5.ZIP › HTML_browsable_motif_output/WaRSwap_ath_tair10_withSelfLoops/sigs_WSWRSLDraw_TAIR10_SLColors-2500.pvals.heatmaps.html/motif_id_102_301130113_tftype_ath_upstream_-3000_0.html]

```
BG_MODEL = WARSwap
MOTIF_ID = 102_301130113
TF_TYPE = ath
UPSTREAM = -3000_0


PVals
FNR = 0.2	FNR = 0.4	FNR = 0.6	FNR = 0.8
deltaG = 60	0.004	0.162	0.592	0.026
deltaG = 70	0.002	0.151	0.605	0.023
deltaG = 80	0.002	0.16	0.606	0.022

ZScores
FNR = 0.2	FNR = 0.4	FNR = 0.6	FNR = 0.8
deltaG = 60	3.895	0.905	-0.743	-0.15
deltaG = 70	4.053	0.953	-0.757	-0.148
deltaG = 80	3.932	0.887	-0.761	-0.141

StDevs
FNR = 0.2	FNR = 0.4	FNR = 0.6	FNR = 0.8
deltaG = 60	15.883	14.085	2.41	0.21
deltaG = 70	15.413	13.678	2.413	0.164
deltaG = 80	15.782	14.037	2.363	0.182
```
